# Stroma Involvement in Pancreatic Ductal Adenocarcinoma: An Overview Focusing on Extracellular Matrix Proteins

**DOI:** 10.3389/fimmu.2021.612271

**Published:** 2021-04-06

**Authors:** Sophie Liot, Jonathan Balas, Alexandre Aubert, Laura Prigent, Perrine Mercier-Gouy, Bernard Verrier, Philippe Bertolino, Ana Hennino, Ulrich Valcourt, Elise Lambert

**Affiliations:** ^1^Laboratoire de Biologie Tissulaire et Ingénierie Thérapeutique (LBTI), UMR CNRS 5305, Université Lyon 1, Institut de Biologie et Chimie des Protéines, Lyon, France; ^2^Cancer Research Center of Lyon, UMR INSERM 1052, CNRS 5286, Lyon, France

**Keywords:** pancreatic ductal adenocarcinoma, stroma, tumor microenvironment, extracellular matrix, tenascin

## Abstract

Pancreatic cancer is the seventh leading cause of cancer-related deaths worldwide and is predicted to become second in 2030 in industrialized countries if no therapeutic progress is made. Among the different types of pancreatic cancers, Pancreatic Ductal Adenocarcinoma (PDAC) is by far the most represented one with an occurrence of more than 90%. This specific cancer is a devastating malignancy with an extremely poor prognosis, as shown by the 5-years survival rate of 2–9%, ranking firmly last amongst all cancer sites in terms of prognostic outcomes for patients. Pancreatic tumors progress with few specific symptoms and are thus at an advanced stage at diagnosis in most patients. This malignancy is characterized by an extremely dense stroma deposition around lesions, accompanied by tissue hypovascularization and a profound immune suppression. Altogether, these combined features make access to cancer cells almost impossible for conventional chemotherapeutics and new immunotherapeutic agents, thus contributing to the fatal outcomes of the disease. Initially ignored, the Tumor MicroEnvironment (TME) is now the subject of intensive research related to PDAC treatment and could contain new therapeutic targets. In this review, we will summarize the current state of knowledge in the field by focusing on TME composition to understand how this specific compartment could influence tumor progression and resistance to therapies. Attention will be paid to Tenascin-C, a matrix glycoprotein commonly upregulated during cancer that participates to PDAC progression and thus contributes to poor prognosis.

## Introduction

Pancreatic cancer is relatively rare and represents 2.5% of all cancers worldwide in 2018 (1). However, the fatal outcome of this disease is almost inevitable which consequently ranks this cancer site as the most devastating one. This poor survival is mainly inherent to the fact that this cancer evolves with few specific symptoms and is therefore mostly diagnosed at an advanced stage when the cancer presents a very aggressive behavior (4). Upon cancer detection, resection is possible in 10–20% of the cases, depending on tumor stage, and localization. Before or after surgery, or for unresectable tumors, various treatments including chemotherapeutic agents (gemcitabine, nab-paclitaxel, 5-fluorouacil, or FOLFIRINOX) and radiotherapy are generally used, but demonstrate little improvement of patient survival (4–6). Therefore, the discovery of new therapeutics and/or earlier detection of the disease before the onset of signs and symptoms is mandatory to improve patient survival rate.

**Figure 1:**
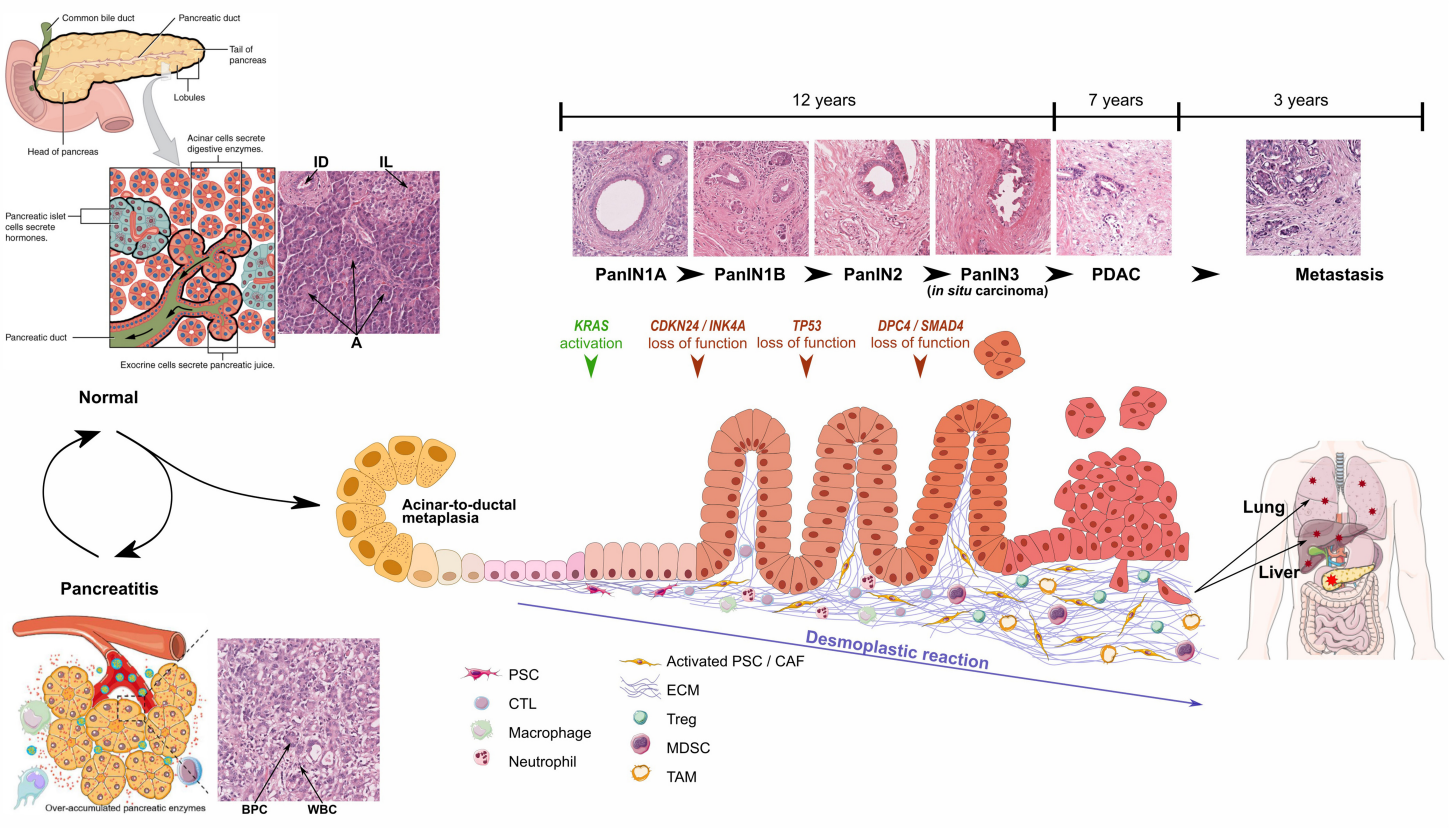
Schematic representations and optical microscopic images of normal pancreas, pancreatitis, and progression from PanIN1 to invasive PDAC. Particular focus is made on development of desmoplastic stroma. Pancreatitis schematic representation from Yao et al. (2), PanIN progression inspired from Morris et al. (3). ID, Interlobular Duct; IL, Islet of Langerhans; A, Acini; BPC, Benign Pancreatic Cells; WBC, White Blood Cells. Healthy pancreas scheme has been obtained from OpenStax College - Anatomy & Physiology. http://cnx.org/content/col11496/1.6/.

Acinar cells are the predominant cell type in the pancreas and present an intrinsic plasticity enabling them to perform metaplasia to ductal-like cells. This metaplastic process called acinar-to-ductal metaplasia (ADM), is observed during acute and chronic pancreatitis and may represent the initial step toward the formation of pancreatic intraepithelial neoplasia (PanIN), which may then progress to PDAC. PanIN lesions are classified in different grades, from PanIN1A to PanIN3, characterized by the evolution of epithelial cell morphology (Figure 1).

Pancreatic carcinogenesis is a multi-stage process resulting primarily from the accumulation of genetic alterations (average of 63 mutations per patient) in the somatic DNA of normal cells as well as inherited mutations (7). Among the numerous referenced alterations, *KRAS, CDKN2A, TP53*, and *SMAD4* are the four most frequently mutated genes. *KRAS* proto-oncogene mutations have been detected in 92% of PDAC and are already detectable in precursor lesions, including early preinvasive intraepithelial neoplasia. Interestingly, *SMAD4* mutations are associated with tumor size, lymphatic invasion, and metastasis and no survival at 5 years (8).

Besides the dramatic modifications in epithelial tissue morphology and genome, PDAC formation is also characterized by the desmoplastic reaction induced by tumor cells, which corresponds to a profound modification of the connective tissue through (1) recruitment and activation of specific fibroblasts and (4) intense ECM deposition. Initially corresponding to around 5% of pancreas mass, the connective tissue thus largely develops up to 90% of tumor area (60% on average) (9). Changes in stroma composition also lead to modifications in local immune system and vascularization, which dramatically influence prognosis (10, 11). However, the TME also contains anti-tumor components, which could explain why strategies depleting connective tissue cells have been so ineffective or even deleterious (12, 13).

The purpose of this review will thus be to describe the changes (1) in the cellular composition of the TME, as well as (4) in the ECM composition by attempting to identify which proteins have a potential pro- or anti-tumoral role with the ultimate aim of bringing out new therapeutic targets.

### Cellular Composition of the Stroma

#### Cancer-Associated Fibroblasts

Among the various PDAC stromal cell types, the Cancer-Associated Fibroblasts (CAFs) are the most abundant. The CAF population presents a high heterogeneity and diversity of functions, presumably due to the multiple origins of these cells (14). Indeed, they can originate from tissue-resident fibroblasts that are activated under the control of growth factors such as TGFβ or following genetic mutations such as TP53 or PTEN (15). Another cellular origin of CAFs, and probably the most frequent one, is the Pancreatic Stellate Cells (PSCs) (16). PSC activation occurs following pancreatic injury, or upon PDGF or TGFβ stimulation, and leads to (1) morphological changes from a star-like shape into spindle-like cells, (4) loss of vitamin-A droplets and (5) increase in cell nucleus volume (17–19). CAFs may also derive from the recruitment and differentiation of bone marrow-derived mesenchymal stem cells or from the trans-differentiation of non-fibroblastic lineages such as adipocytes or epithelial cells (12, 20, 21). Various markers can be used to distinguish the multiple subsets of CAFs, such as PDGF-receptor α and β (PDGFRα/β), α-SMA, FAP, and S100A4 (Ca^2+^-binding protein), but none of them is exclusively expressed by CAFs, further highlighting CAF heterogeneity (21, 22). CAFs are responsible for the deposition of a dense tumor stroma, which subsequently can function as a physical barrier against immune infiltration or as a structural scaffold for cell interactions. In addition, CAFs secrete MMPs, which consequently ensure ECM degradation and the subsequent release of various factors leading to the recruitment of specific cells and/or cell dissemination. Finally, CAFs also produce many growth factors and proinflammatory cytokines such as TGFβ, vascular endothelial growth factor (VEGF), interleukin-6 (IL-6) and CXC-chemokine ligand 12 (CXCL12), thereby promoting tumor growth, angiogenesis and recruitment of immunosuppressive cells into the TME to assist in immune evasion (20, 23). In an effort to depict the fibroblast heterogeneity, 3 different CAF subpopulations have been identified according to their function or gene signature: the “inflammatory,” “myofibroblastic” and “antigen-presenting” CAFs (24, 25).

#### Endothelial Cells

Despite the obvious production of pro-angiogenic factors by CAFs and pancreatic tumor cells, PDAC is characterized by a low microvascular density compared to other types of cancers (11). Indeed, the dense fibrotic stroma forms a physical barrier that inhibits the formation and the proper functioning of vasculature, resulting in sparse constricted blood and lymphatic vessels that are only partially functional and physically separated from the cancer cells. This feature is deleterious for patient survival since low vascularity is associated with poor patient survival due to poor anti-cancer immune cell infiltration and chemotherapeutic drug delivery (26). Consequently, addition of anti-angiogenic drug (bevacizumab) to standard chemotherapy demonstrated no improvement in PDAC outcome (27). On contrary, vascular normalization aiming at improving drug delivery could be a good strategy for this type of carcinoma (27).

#### Infiltrating Immune Cells

Chronic pancreatitis is a risk factor for the development of PDAC as well as of systemic diseases characterized by chronic low-grade inflammation, such as metaflammation in patients with the metabolic syndrome or diabetes (28, 29). Interestingly, chronic pancreatitis and PDAC tissues show similarities in their desmoplasia and inflammatory infiltrates, indicating overlapping inflammatory responses.

The prevention and elimination of cancer cells are dependent on the immune system around the tumor. The PDAC immune microenvironment is characterized by (1) the exhaustion of anti-cancer immune cytotoxic T lymphocytes notably due to high mechanical constraints within the tumor and (4) the infiltration of multiple types of tumor-promoting immune cells, including myeloid-derived suppressor cells, tumor-associated macrophages and regulatory T cells (10, 30). Those tumor-promoting immune cells, in combination with CAFs and cancer cells secrete various pro-inflammatory cytokines such as TGFβ, TNFα, and different interleukins which subsequently favor immune evasion, PDAC development and metastasis formation (31). Various strategies are currently developed to treat PDAC by restoring proper immune system function: enzymatic digestion of TME, vascular normalization and neutralization of immune system modulators (21).

### ECM Evolution During Pancreatic Carcinogenesis

PDAC is characterized by an intense desmoplastic reaction, defined as the fibrotic response of healthy tissue to invasive carcinoma and consisting of an abnormal accumulation of ECM components, mostly collagen fibers (32). This new TME acts as a physical barrier preventing (1) proper angiogenesis, and subsequent drug delivery, and (4) anti-cancer immune infiltration (33, 34). Consequently, TME has been considered as deleterious for patient prognosis and CAFs, which are responsible for dense ECM deposition, have been the target of clinical trials. However, CAF depletion resulted in an apparent paradoxical accelerated disease progression and encouraged a more detailed analysis of the most differentially regulated ECM components in the pancreatic tumors vs. healthy tissue, in order to identify new therapeutic targets within the TME (35, 36). We hereafter describe these matrix components according to the matrisome classification [Table 1; (37)].

**Table 1:**
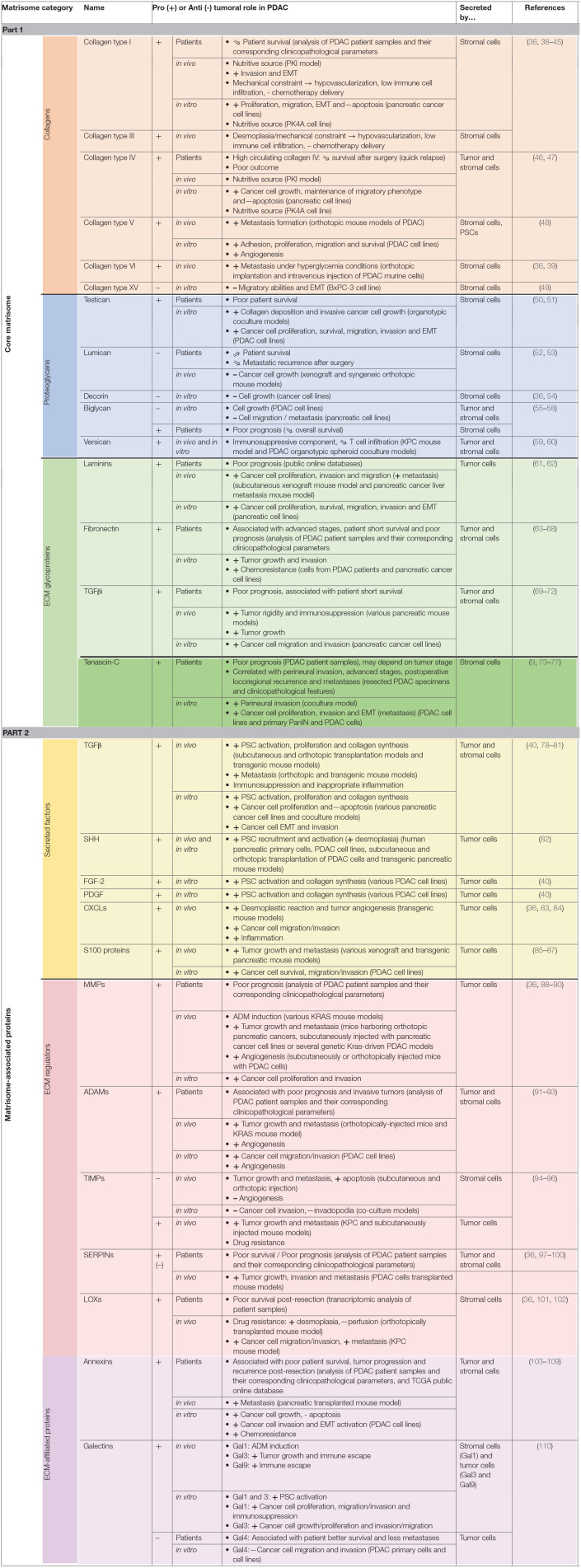
**ECM proteins involved in PDAC, presented according to the matrisome classification.**.

*Pro-(+) or anti-(–) tumoral role as well as cells responsible for their secretion are detailed. **+**: promotion, **–**: inhibition, ⇒ : increased, ⇒ : decreased. Each protein family has been classified according to the matrisome classification and is highlighted with a specific color. Tenascin-C information is highlighted in dark green*.

#### Core Matrisome

##### Collagens

Collagens are by far the most represented constituents of the connective tissue of normal and pathological pancreas (>90% of ECM proteins), with the type I and III fibrillar collagens accounting for >90% of all collagen mass (36, 38). Protein level for those collagens increases 2.6-fold during pancreatic tumor progression, which explains desmoplasia and justifies them as crucial targets. Additionally, the stroma undergoes intense rearrangement, leading to highly aligned collagen fibers, associated with bad prognosis for patients following pancreatic cancer resection (111). Despite their increased deposition, no ratio variation is observed for type I and III collagens between healthy and PDAC connective tissues, thus encouraging attention to other collagens differentially expressed during pancreatic carcinogenesis (37). Among them, type IV, V, VI, VII, XII, XIV, and XV collagens are key players in pancreatic tumorigenesis and act either as beneficial or detrimental molecules. For instance, collagen IV, which is an essential constituent of the basement membrane (BM), is produced by cancer cells, favors cancer cell growth, migration and protect them from apoptosis. Consequently, high serum level of collagen IV is associated with quick relapse after surgery and thus poor survival (46, 47). On contrary, another BM component, collagen XV, is lost during pancreatic tumorigenesis and its overexpression reduces the migratory abilities of PDAC cells in type I collagen-rich matrices (49). Interestingly, collagen VI is highly expressed during PDAC progression, induces metastatic colonization particularly in a hyperglycemic context and could therefore be targeted especially in diabetic patients (36, 39).

Besides their architectural and signaling role enabling tumor progression, collagens also serve as a nutritive source. Indeed, under PDAC specific conditions low in oxygen and nutrients, tumor cells metabolize collagen molecules, and thus collagen-derived proline enables PDAC cell proliferation (112). Therefore, this could explain the correlation between high serum collagen fragment levels in serum and significantly shorter overall survival, and prompts detailed analysis of collagen fragment role during PDAC progression (113).

##### Proteoglycans

Proteoglycans consist of one or more glycosaminoglycan (GAG) chain(s)—representing around 85% of the molecule mass—covalently attached to a core protein, and are categorized depending of their GAG chain nature and size (114). Among the small proteoglycans which are mainly expressed by TME, testican acts as a pro-tumoral molecule by affecting collagen deposition and thus favoring tumor cell growth and invasion, therefore leading to a poor patient survival, whereas lumican interferes with tumor progression and is associated with prolonged patient survival by limiting cancer cell growth and metastasis (50, 52, 53). Decorin is also considered as an anti-tumoral constituent by reducing tumor cell growth (36, 54) whereas the pro- or anti-tumoral status of biglycan in PDAC is still under debate. Indeed, its expression by stromal and epithelial cells (1) is induced by TGFβ, (4) has been described to inhibit pancreatic cancer cell growth and migration (55–57), but (5) is associated with poor prognosis (58). Finally, versican, which corresponds to a large proteoglycan expressed by both stromal and epithelial cells in PDAC, acts as an immunosuppressive component by reducing T cell infiltration and is thus considered as a deleterious molecule for patient survival (59).

##### Glycoproteins

Laminins are a family of ECM glycoproteins representing the major non-collagenous constituent in BM. Most of their subunits are over-expressed in PDAC and associated with poor outcome for patient survival (61). Fibronectin (FN1), which supports cell-ECM interactions, is essential for wound healing, development, and tissue homeostasis under physiological context. FN1 is also upregulated in PDAC which leads to tumor growth, invasion and metastasis formation and is consequently associated with poor prognosis in PDAC patients (63).

Transforming Growth Factor beta-induced (TGFβi) protein, also named βig-h3, is able to modulate cell adhesion through various integrins, including αvβ3, α1β1, and αvβ5.This glycoprotein is increased during pancreatic cancer and acts either directly on tumor CD8^+^ T cells by reducing their proliferation and activation or on tumor cells by promoting their migration and invasion (69, 115). TGFβi could thus be regarded for its double therapeutic potential to increase local anti-tumor immunity and subsequently induce cancer cell apoptosis or inhibit metastasis (69–72).

Among the four members of the Tenascin (TN) family, Tenascin-C (TNC) is by far the most well-characterized and is commonly described as being widely distributed in embryonic tissues, restricted in some adult tissues, such as stem cell niches and tendons (116), and *de novo* re-expressed during physio-pathological contexts such as wound healing and tumor progression (117). In PDAC, TNC protein is restrained to the tumor stroma and is not found in epithelial tumor cells or adjacent normal pancreatic tissue (9). High TNC expression, and downstream signaling through the Annexin II receptor, have been initially correlated with poor prognosis but this association is still controversial and could depend on the stage and grade of the pancreatic tumor or the specific location of TNC (9, 73, 118). So, high perineural TNC expression is associated with perineural invasion and poor prognosis with high loco-regional recurrence (74). In the same line, a recent study highlighted TNC as a prominent protein found in exosomal compartment and associated with local invasion and distant metastasis (75).

We recently demonstrated *TNXB* gene and TNX protein were significantly downregulated in the six cancers with the highest incidence and mortality worldwide (i.e., lung, breast, prostate, stomach, colorectal, and liver carcinomas) and low TNX levels were associated with poor prognosis in patients suffering from lung and breast carcinomas (119). In the same study, TNX protein expression was also decreased in tumor samples from PDAC patients (119).

#### Matrisome-Associated Proteins

##### Secreted Factors

In pancreatic cancer, Various Factors Are Mainly produced by cancer cells to favor tumor progression. Among them, TGFβ role is complex and mediates both pro- and anti-tumoral activities in cancer cells depending on their context, in space and time and their microenvironment. Indeed, in normal pancreatic cells and at early stages of pancreatic carcinogenesis, TGFβ exerts a tumor suppressive effect through SMAD4-regulated genes. However, in the late phase, SMAD4 is inactivated whereas TGFβ expression is upregulated leading to PI3K/Akt, Ras/ERK, p38MAPK, and Rho/GTPase pathway activation and subsequent tumor progression (120). Then, TGFβ invariably induces (1) proliferation and survival of PDAC cells, (4) EMT, invasion, and metastasis, (5) production of a dense fibrotic stroma and (6) deregulation of the immune microenvironment toward immunosuppression and inappropriate inflammation. Thus, various promising pre-clinical and clinical trials have already evaluated the potential of TGFβ-targeting therapies, through TGFβ regulator (losartan), TGFβ neutralizing antibodies or TGFβ receptor inhibitors (78–81, 121, 122). Other signaling factors are secreted by pancreatic cancer cells, enabling PSC recruitment and activation, and subsequent desmoplastic response inducing collagen synthesis. Thus, Sonic HedgeHog (SHH), Fibroblast Growth Factor-2 (FGF-2) and Platelet-derived Growth Factor (PDGF) are overexpressed during PDAC and interfering with their signaling corresponds to valuable strategies for PDAC treatment (40, 82). However, clinical trials using IPI-926, vismodegib and sonidegib that target the hedgehog pathway have so far been disappointing (123). PDAC cells also overexpress several CXC ligands, which are involved in desmoplastic reaction, immune modulation and tumor angiogenesis (36, 124). Thus, blocking the CXCLs-CXCR2 axis improves survival in a PDAC developing mouse model by reducing cell invasion and inflammation and could be a therapeutic approach against PDAC progression (83, 125). Finally, proteomic analyses of ECM during PDAC progression demonstrated that various members of the S100 Ca^2+^-binding protein family, notably S100P and S100A4, are upregulated in this disease and their high levels are associated with poor prognosis, thus shedding light on their receptor, i.e., the Receptor for Advanced Glycation End products (RAGE) and the RAGE/S100 ligand axis as a promising therapeutic approach (85). Therefore, various S100 monoclonal antibodies, S100 protein inhibitors or RAGE antagonist have already demonstrated a reduction of tumor growth and metastasis formation in mouse models (85).

##### ECM Regulators

Many proteins overexpressed during pancreatic tumorigenesis are responsible for ECM remodeling and are therefore potential targets for pancreatic cancer treatment. Matrix MetalloProteinases (MMPs), A Disintegrin And Metalloproteinases (ADAMs), and A Disintegrin And Metalloproteinase with ThromboSpondin motifs (ADAMTSs) are zinc-dependent endopeptidases that are able to degrade all ECM proteins. Their activities are tightly regulated by proteolytic activation and inhibition *via* their natural inhibitors, Tissue Inhibitors of MetalloProteinases (TIMPs) (126). The imbalance between the expression of metalloproteinases and TIMPs is thus of crucial interest in cancer development and metastasis (127). With some exceptions, those proteases are overexpressed during pancreatic cancer progression and are the targets of numerous pre-clinical and clinical trials, which for some of them were disappointing or less powerful than expected, probably due to (1) aspecific metalloproteinase targeting (use of broad-spectrum inhibitors) or (4) compensation mechanisms set up by tumor cells (88).

Several SERPIN family members are also importantly differentially regulated during PDAC development, mainly promoting tumor growth, invasion, and are associated with poor survival, but their activities have to be analyzed individually with particular attention paid to their original cells (36, 97–99). Finally, Lysyl Oxidases (LOX), a family of extracellular copper-dependent enzymes involved in ECM cross-linking, are also important matrix regulators over-represented during pancreatic tumorigenesis (36). Their inhibition in mouse model prolonged tumor-free survival by interfering with stroma stiffness (101, 102).

##### ECM-Affiliated Proteins

Among the ECM-affiliated proteins significantly deregulated during PDAC development, numerous members belong to the vertebrate “A subgroup” of the annexin superfamily coding a calcium- and membrane-binding protein (36). This subgroup consists of at least 12 members (A1-A11 and A13), all of which are suspected to be involved in tumor development (103). In PDAC, annexins are known to favor tumor cell growth, EMT, invasion and metastasis and to inhibit apoptosis. Additionally, they interact with various peri-cellular proteins such as S100 proteins and TNC, which are upregulated during PDAC progression. Therefore, annexin overexpression is associated with poor patient prognosis and could inspire new therapeutic strategies (128). Galectins, which are a family of carbohydrate-binding proteins, are also upregulated during PDAC progression (36, 110). Besides galectin-4, which has been described as a tumor suppressor by inhibiting tumor cell migration and invasion, the other galectins favor pancreatic tumor. Consequently, galectin inhibitors are considered as promising opportunities for pancreatic cancer therapeutic interventions, either alone or combined with current chemo- and/or immunotherapies (110).

## Conclusions

During pancreatic tumorigenesis, important stromal modifications occur both at the cell landscape level and the matrix molecular composition in response to tumor signals. Herein, we have listed these major changes by focusing only on the proteins belonging to the matrisome. However, other extracellular components have not been underlined, but can drastically influence pancreatic tumor progression, as it is the case for hyaluronic acid (HA) (129–136). Indeed, in PDAC mouse model, HA deposition (1) was observed very early during tumor formation in an intralobular position in ADM regions and close to PanIN lesions and (4) preceded collagen deposition around lesions that will progress to PDAC (129). Besides making the ECM denser, HA deposition may be related to an inflammatory stage allowing the recruitment of immune cells in ADM areas, which further underlines the value of HA as a therapeutic target for PDAC treatment. Various drugs targeting HA have been developed such as pegylated hyaluronidase (PEGPH20) and Minnelide (132, 137). However, PEGPH20 in combination with conventional chemotherapies failed to demonstrate an improvement in median overall survival, leading to the recent discontinuation of PEGPH20 development after a phase 3 clinical trial. Minnelide, corresponding to an active substance extracted from thunder god vine is still under investigation and its mechanism of action seems multimodal (132, 138–140). Additionally, abnormal glycosylation of ECM components such as proteoglycans and glycoproteins can significantly influence tumor growth, neoplastic progression, metastasis and chemoresistance and thus should be considered for new drug design (141, 142). So far, despite promising results in preclinical models, no therapeutic strategy targeting the stroma compartment has brought conclusive results in clinical settings. This could be explained by differences in pharmacokinetics, pharmacodynamics and metabolism and the failure to accurately model the tumor microenvironment of patients using preclinical mouse models. However, a better understanding of the tumor stroma is expected to open up new possibilities for the development of new drugs.

## Author Contributions

SL and JB: data curation, investigation and writing—original draft. AA, LP, and PM-G: writing—review and editing. BV: funding acquisition. PB and UV: writing—review and editing and funding acquisition. AH: investigation and writing—review and editing. EL: conceptualization, supervision, and writing—original draft. All authors: contributed to the article and approved the submitted version.

## Conflict of Interest

The authors declare that the research was conducted in the absence of any commercial or financial relationships that could be construed as a potential conflict of interest.
